# Adult attention-deficit hyperactivity disorder: Why should we pay attention?

**DOI:** 10.4102/sajpsychiatry.v23i0.1072

**Published:** 2017-08-31

**Authors:** Renata Schoeman, Ruth Albertyn, Manie de Klerk

**Affiliations:** 1 Stellenbosch University Business School, Stellenbosch University, South Africa; 2 Private Practice, Cape Town, South Africa; 3 MMI Health Centre of Excellence, South Africa

## Abstract

**Background:**

Attention-deficit hyperactivity disorder (ADHD) is a common neurodevelopmental disorder, with a chronic, costly and debilitating course if untreated. Limited access to diagnosis and treatment for adults with ADHD contributes to the cost of the disorder and the burden of disease.

**Aim:**

This study aims to identify the barriers to care for adults with ADHD.

**Methods:**

A qualitative analysis of semi-structured interviews with 10 key opinion leaders in the field of adult ADHD in SA was conducted to obtain narratives regarding frustrations experienced when treating adults with ADHD and needs of patients regarding management of ADHD. Qualitative content analysis was completed using Atlas.ti (version 7).

**Results:**

Four key themes which emerged from the interviews were ‘lack of recognition of the disorder’, ‘lack of access to diagnosis’, ‘lack of access to treatment’ and ‘a life of perpetual failure’. Core to these themes are the lack of knowledge amongst health care professionals, funders, and society at large.

**Conclusion:**

Our findings expand on previous research regarding the need to increase the knowledge base on adult ADHD. A collaborative stakeholder approach is needed to provide research and training for improved diagnosis and treatment for adults with ADHD in the South African context.

## Introduction

Attention-deficit hyperactivity disorder (ADHD) has received increased scientific, clinical and public attention over the past few decades and is now recognised as a common neurodevelopmental disorder, present throughout life, with a chronic, costly and debilitating course if untreated. Unfortunately, many patients suffering from ADHD remain undiagnosed, or if diagnosed, they do not receive optimal treatment.

Improved outcomes are possible if patients suffering from ADHD receive a timely and accurate diagnosis, and receive comprehensive care, which would include psychopharmacological interventions, behavioural interventions and support. Previous research established the knowledge gap amongst general practitioners (GPs) with regard to adult ADHD. ^1^ In South Africa (SA), poor identification and treatment of common mental disorders at primary healthcare level, limited access to specialist resources and a large service delivery and treatment gap remain problematic. In the current environment of a pressured health care system, GPs will play an increasingly important role in the early identification and management of adults with ADHD. ^2,3^

In this article, we argue that unless the lack of knowledge with regard to adult ADHD is addressed, the barriers to access to diagnosis and treatment will continue to prevail. Strategies to break down these barriers include research, education, information and communication through multiple stakeholder engagement.

## Literature review

The notion that ADHD is a disorder of childhood prevailed until the 1990s. However, rigorous research, including longitudinal studies, has now firmly established the existence of adult ADHD. ADHD affects 2% – 16% of the school-age population.^4^ Estimates of the prevalence of adult ADHD range between 2.5% and 4.3%.^5,6^ Extrapolating from population statistics (based on StatsSA population statistics for SA) and known prevalence information, we calculated the expected number of adults in SA between the ages of 20 and 50 affected by ADHD to be around 1 million. In the first South African study exploring the situation with regard to the prevalence and treatment of adult ADHD the population prevalence of adult ADHD was estimated at 1.09%, while the prevalence in clinical psychiatric settings was as high as 52.5% (of which 13.68% were patients with newly diagnosed ADHD).^7^ This is a conservative estimate and lower than international prevalence rates and may be attributed to a lack of awareness and knowledge amongst health care practitioners (HCPs) – which hampers the diagnosis of patients.

As from 01 January 2015, psychiatrists in SA use the *Diagnostic and Statistical Manual for Mental Disorders*, 5th edition (DSM-5) criteria for the diagnosis of ADHD.^8^ Core symptoms of ADHD include a persistent pattern of inattention and/or hyperactivity-impulsivity that interferes with functioning or development of the individual. The DSM-5 relaxed the diagnostic criteria for ADHD with the onset of symptoms specified as before 12 years of age and individuals older than 17 years are required to meet fewer criteria (5+ symptoms).

The core symptoms of ADHD often appear to decrease over time – in number and in severity. However, adults are often more adept at managing these symptoms. Some adults compensate for ADHD-related impairment by choosing lifestyles and careers that suit them. Although they may appear to function well, excessive amounts of energy and time are used to accomplish tasks. Associated symptoms of ADHD include behavioural, cognitive, emotional and social problems. Problems with planning, task-initiation, task-completion, impatience and impulsivity can cause numerous work-related and interpersonal problems. This impairs educational attainment and work performance (reduced productivity and increased absenteeism), and add to significant societal costs (e.g. due to substance abuse and accidents) – impacting negatively on overall quality of life.^9,10,11^ Individuals with untreated ADHD, their families and other caregivers must be made aware of the impact that this disorder may have on them at every stage of life and, correspondingly, the improved outcomes that can be achieved with the successful management of ADHD. The presence of adult ADHD increases the prevalence of multiple medical and psychiatric comorbidity (estimated at more than 50% in adults with ADHD), and doubled the healthcare costs of individuals^7,12,13^

Many studies have been conducted and established the efficacy and effectiveness of both stimulant- and non-stimulant medications in the treatment of ADHD in children and adolescents, and more recently also in adults. Pharmacotherapy plays a primary role in the treatment of ADHD, but psychosocial interventions (psycho-education, cognitive behavioural therapy, supportive coaching or assistance with daily activities) form an integral part of management.^14,15,16^

## Ethical considerations

Approval for the project was obtained from the University of Stellenbosch Business School Ethics Committee.

## Methods

A qualitative analysis of semi-structured interviews with key opinion leaders (KOLs) in the field of adult ADHD in SA was conducted. This study formed part of a larger triangulated study (consisting of a retrospective claims database analysis, a survey and this in depth interviews component) in which the current situation in SA with regard to the prevalence, diagnosis and management of adult ADHD was explored. The aim of the interviews was to elicit narratives of these experts working with patients with adult ADHD.

### Key opinion leaders: Inclusion criteria

Our sample consisted of 10 experienced specialists, nominated by peers and professional bodies, and identified as KOLs according to specified criteria.^17^ The Mental Health Information Centre (MHIC), Health Professions Council of South Africa (HPCSA) and Psychiatry Management Group (PsychMG) were contacted to establish who are seen as the current KOLs in adult ADHD in South Africa. The definition of KOLs was provided to these bodies based on the definitions of Health Connexions and PRWeb, that KOLs are the people within a group who are the most influential. They influence their peers’ medical practice by speaking at conferences, publication, prescribing habits, establishing protocols, advocacy, training and marketing feedback.^18,19^ In addition, possible KOLs were also identified from the responses from the 103 psychiatrists who participated in the survey leg of the triangulated study. All 10 KOLs contacted provided consent for participation.

### Data collection: Recording of interviews

Semi-structured interviews (telephonic or in-person) were conducted between February 2014 and April 2014. Three open-ended questions were asked, with probes where needed to extend the answers provided without leading the desired response, with regard to the frustrations experienced when treating adults with ADHD, the needs the patients have with regard to management of ADHD, and suggestions and solutions for better management of these patients. All interviews were recorded once respondents had given permission for the recording as specified in the ethical procedures for this study.

### Data analysis

Recorded interviews were transcribed by an external professional (who was also bound to confidentiality), checked for accuracy by R.S., followed by qualitative content analysis of the data by means of Atlas.ti (version 7), a Computer Aided Qualitative Data Analysis Software (CAQDAS) programme.^20^

A framework approach was followed in which the objectives of the investigation were set in advance. ^21^ Although this approach reflects the original accounts and observations of the people studied (an inductive process), it starts deductively from pre-set aims and objectives (subcategories were built based on items within the main category). The datasets were read to form an overview and to capture the context in which the data exist. Hereafter, dual descriptive level (level one open-coding, where segments of meaning were identified and level two free-coding) and conceptual-level analysis (where related codes were categorised into groups and where relationships between categories were sought to identify themes) followed. Data saturation was sensed by interview 7.

In order to protect against researcher bias, the researcher (R.S.) coded all the data and then gave the entire set to an independent analyst (R.A.) to check – eight of which were already coded. The analyst then independently coded the remaining two of the 10 interviews. The researcher and independent analyst met to compare and discuss their coding and used the feedback to ensure the quality of the set of interviews and analysis thereof.

The identified KOLs were contacted and consent for interviewing them, and for the recording of the interviews, was obtained. Data was not linked to personal identifiers.

## Results and discussion

The KOLS interviewed were mostly male (90%) and represented the key urban centres (Western Cape, Gauteng, KwaZulu-Natal, the Eastern Cape and the Free State), where most psychiatrists are located.

The final code list consisted of 219 codes, representing 1011 quotes which were grouped into 24 families: acceptance, access to care, adequate treatment, awareness, chronic condition, comorbidity, complications, comprehensive care, costs, diagnosis, education, family, financial impact, funding, impulse control problems, knowledge gaps, medical schemes, medication, misconceptions, psychotherapy, regulation, stigma, training and work environment.

**FIGURE 1:**
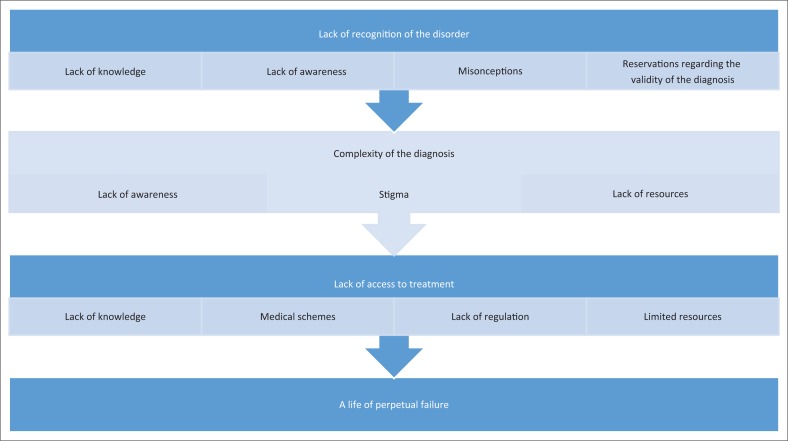
Key themes emerging from the interviews.

The four key themes which emerged from the interviews are presented (Figure 1). Core to these themes are the lack of knowledge amongst health care professionals, funders and society at large. Quotes by participants are indicated as, for example, (P1) for participant 1.

### Lack of recognition of the disorder

Various factors contribute to the lack of recognition of adult ADHD as a diagnostic entity. There are misconceptions amongst the general public surrounding ADHD, such as the addictive nature of medication (P7, P8 and P9), ADHD as an scholastic or academic problem only (P6, P8), ADHD as a disorder of childhood (P2, P3, P4 and P7) and the perception that it is over diagnosed because of financial motives of psychiatrists and pharmaceutical companies (P4 and P7). These misconceptions are further enforced by antipsychiatry groups and incorrect media information (P7 and P8).

A lack of awareness and knowledge, as well as misconceptions surrounding the diagnosis amongst HCPs and allied HCPs, leaves them unable to have an informed opinion or to psychoeducate patients and the community. Many HCPs received no training on ADHD and even less so on adult ADHD:

‘It’s not something that you’re taught about, and it’s sometimes actually even a bit of frowned upon – that it doesn’t exist or this sort of thing.’ (P7)

Although the diagnostic criteria for ADHD have been validated, many HCPs still challenge the relevance and appropriateness of these criteria in the adult population (P3, P4, P6, P9 and P10). This further contributes to the controversy surrounding the diagnosis. This lack of knowledge creates a cloud of ignorance surrounding ADHD, which further contributes to the stigma of the disorder and to psychiatry in general as illustrated by the following comment:

‘There is always lots of convincing the patient (author clarification: lots of effort in convincing the patient), and you have to convince everyone else: the funders, the colleagues, the general practitioners. There is a lot of resistance to the diagnosis.’ (P6)

### Lack of access to diagnosis

Although there is a perception that adult ADHD is over diagnosed, KOLs reported that this is not the case (P4, P7). Due to a lack of awareness of the disorder, as well as the stigma surrounding the diagnosis and psychiatry in general, many patients suffering from ADHD never present to psychiatrists for diagnosis (P3 and P10):

‘I think that the adults often would come to me, and they would be reluctant to disclose some of their symptoms because they don’t know whether it is really an illness, and they are not always happy to disclose to the workplace or other people that they indeed have adult ADHD.’ (P1)

ADHD is a chronic disorder with a differential presentation across the lifespan, for example, hyperactivity which becomes less evident, while interpersonal, financial and work related problems become more evident (P3, P4 and P5). Comorbidity such as anxiety disorders, mood disorders and substance use disorders (P1, P2, P3, P5, P6, P7, P8 and P9), or complications such as marital distress or driving accidents (P1, P2, P3, P4 and P8) often are the presenting problems. ADHD requires a comprehensive assessment (P1, P4 and P5), including collateral information. If a HCP do not specifically address this history, the diagnosis of ADHD will be missed, or a patient will be misdiagnosed as suffering from another disorder (P5, P6 and P7). A lack of resources – both in terms of manpower (access to skilled health care professionals with adequate and comprehensive training, an interest in the disorder, and well-versed in the diagnostic challenges and subtleties of the disorder) and finances (transport and consultation fees) can further impede the chances of receiving a correct diagnosis (P10). In addition to difficulties with access to diagnosis and treatment, is the acceptance of the patient of suffering from a chronic disorder, and therefore compliance with treatment (P6, P8 and P9).

### Lack of access to treatment

KOLs reported that, even when a patient has been diagnosed with adult ADHD, they often do not have access to treatment (P2, P5, P7 and P8) – the ideal being a comprehensive, multidisciplinary approach (P3, P4 and P9) which includes access to medication, psychotherapy (P1, P4, P5, P6, P8, P9 and P10), occupational therapists (P3, P5 and P9) and work intervention (P4, P5 and P9). Medication options are limited in SA (P5) and the cost thereof high (P4 and P9). Medical schemes do not acknowledge the existence of the disorder in adults (P3, P4 and P6) and often discontinue funding for medication as soon as a child leaves school or turns 18 years old (P2 and P6) – attesting to the lack of knowledge of adult ADHD. This hampers continuous treatment, as well as compliance (P4 and P8).

Psychotherapy (both individual and family therapy), life coaching and assistance with time management, organisational skills, and support within the work environment (e.g. by liaison between an occupational therapist and the employee) is virtually inaccessible – again both because of a lack of manpower and financial resources. Late diagnosis and inadequate treatment increase the complications and comorbidities associated with adult ADHD (P1, P8 and P10). Although the direct costs of ADHD are high, these indirect and hidden costs to society are much higher (P3, P4, P6 and P8).

### A life of perpetual failure

Due to a lack of awareness of the disorder in the community, a lack of knowledge by HCPs and allied HCPs, and therefore a lack of access to diagnosis and treatment, many patients live a life of perpetual failure:

‘If you look back into their life, then you can see the chequered work record, relationship failures, the substance abuse, the disruption in their life, the poor financial management over time and the co-morbidities that at interfere over time. That is an enormous cost for that person and for their families.’ (P4)

Adults with ADHD are often aware that they do struggle, but do not know why (P1). Poor work records (P3, P4, P5 and P8), interpersonal (P2 and P3) and marital difficulties (P1, P3, P6, P8, P9), psychiatric and medical comorbidity and complications (P1, P2, P3, P7, P8 and P9) and impulsivity with even forensic consequences (P1, P4, P6, P8 and P9) often mark there life trajectory. Families are frustrated and fatigued because of interpersonal problems and financial difficulties – both because of untreated ADHD, but also because of the financial impact of treatment (P2, P3, P5, P6, P7, P8 and P9):

‘It’s not just academic performance that’s influenced by ADHD, but actually the general life – the wellness of life – that is influenced by the symptoms of ADHD.’ (P8)

## Strategies to break down barriers to care

### Focus areas

#### Access to information

The biggest exhaustive need for patients, families, HCPs, medical schemes and the community at large is access to information on ADHD. It seems that that a lack of knowledge and information feed into misconceptions and stigma about the disorder, and the delayed, missed or misdiagnosis. Informed primary HCPs are crucial in the pathway to care. Clinical strategies to increase knowledge of HCPs include the development of guidelines and pre- and postgraduate training content. A public awareness campaign to increase awareness of both the public and HCPs should be driven by SASOP Special Interest Group for adult ADHD.

#### Access to funding

Lack of funding and costs are barriers to treatment which limit access to care and treatment. ADHD is a chronic, costly and debilitating disorder when left undiagnosed and untreated. One of the reasons for lack of funding by medical schemes includes the lack of recognition of adult ADHD as a disorder and a lack of economic studies. ^7^ This limits access to optimal comprehensive treatment, adds to the direct and indirect costs of ADHD and the emotional and financial burden of the disease. It is therefore crucial to extend the knowledge base from HCPs to medical funders. It is also crucial for KOLs to have input into benefit design in order to gain funding for the health insurance cover of ADHD.

#### Access to a basket of care

The basket of care for adult ADHD has two handles: information and funding. A comprehensive diagnostic assessment can be considered the base, while the post-diagnostic management forms the sides of the basket – containing the patients and their families through a multidisciplinary and multimodal approach.

### Recommendations

The following specific recommendations are suggested:

- Screening and early detection by primary HCPs.
- Increasing the awareness of adult ADHD and its ramifications, as well as training of HCPs in the diagnosis and treatment thereof, can facilitate early detection and timely referral. This can have a positive influence on the trajectory of the disease and of the person’s life.
- Referral to a psychiatrist (or other adequately trained and skilled HCP) for a comprehensive diagnostic assessment.
- As adult ADHD is diagnostically complex, and comorbidity and complications are rife, an initial diagnostic assessment by a psychiatrist is recommended, especially with regard to the presence, severity and treatment of comorbid conditions. A thorough and comprehensive assessment, and a standardised way of making the diagnosis, could also enhance access to treatment through medical scheme funding. Comprehensive training in diagnosis and treatment of these potentially complex patients are crucial to ensure management by skilled and confident HCPs. This will enhance long term outcomes for these patients.
- Comprehensive multi-modal and individualised care.
- Ongoing access to consultations for acute stabilisation, maintenance, and relapse prevention are needed. This should include medication management, psycho-education (for the patient, their families and employers), therapy (psychotherapy and occupational therapy) and support (e.g. coaching and workplace intervention). Once a patient has been stabilised, further management can be at GP level.
- Advance the body of evidence through research (e.g. clinical trials and health economic research).

## Conclusion

Although adult ADHD is now established as a disorder abroad,^8^ based on our interviews with 10 KOLs in SA, there seems to be a lack of awareness thereof and the stigma surrounding psychiatry are often a barrier for patients to access diagnoses. Furthermore, a lack of awareness amongst HCPs, their own misconceptions regarding diagnosis, and lack of knowledge of the diagnosis and treatment hampers the diagnosis, even when patients present to the HCP.

Our findings highlight and expand on previous research regarding the need of GPs (as the gatekeepers to mental health services) to increase their knowledge base with regard to adult ADHD.^4^ Our findings form a foundation to break down the barriers limiting access to diagnosis and treatment for individuals with adult ADHD. It is therefore crucial to improve access to knowledge – through research and training (at university level and through continued professional development activities) – and to embark on a collaborative approach between stakeholders to provide optimal care for adults with ADHD in the South African context.

## References

[1] Louw C, Oswald MM, Perold MD General practitioners’ familiarity, attitudes and practices with regard to Attention Deficit Hyperacivity Disorder (ADHD) in children and adults. SA Fam Pract. 2009;51(2):153–157.

[2] Petersen I, Lund C Mental health service delivery in South Africa from 2000 to 2010: One step forward, one step back. S Afr Med J. 2011;101(10):751–757. 22272856

[3] Seedat S, Stein DJ, Herman A, et al 2008. Twelve-month treatment of psychiatric disorders in the South African Stress and Health study (World Mental Health survey initiative). Soc Psychiatr Psychiatr Epidemiol. 2008;43(11):889–897. 10.1007/s00127-008-0399-9 PMC3222914 18677573

[4] National Resource Centre on AD/HD Statistical prevalence of ADHD [homepage on the Internet]. 2013 [cited 2013 Mar 10]. Available from: http://www.help4adhd.org/en/about/statistics

[5] Fayyad J, De Graaf R, Kessler R, et al Cross-national prevalence and correlates of adult attention-deficit hyperactivity disorder. Br J Psychiatry. 2007;190:402–409. 10.1192/bjp.bp.106.034389 17470954

[6] Simon V, Czobor P, Balint A, Mezzaros A, Bitter I Prevalence and correlates of adult attention-deficit hyperactivity disorder: Meta-analysis. Br J Psychiatry. 2009;194:204–211. 10.1192/bjp.bp.107.048827 19252145

[7] Schoeman R A funding model proposal for private health insurance for Adult Attention-Deficit/Hyperactivity Disorder in the South African context [unpublished Master’s in Business Administration thesis]. Cape Town: Stellenbosch University; 2015.

[8] American Psychiatric Association (APA) Diagnostic and statistical manual of mental disorders. 5th ed. Washington, DC: American Psychiatric Association; 2013.

[9] Mannuzza S, Klein RG, Bessler A, Malloy P, Hynes ME Educational and occupational outcome of hyperactive boys grown up. J Am Acad Child Adolesc Psychiatry. 1997;36(9):1222–1227. 10.1097/00004583-199709000-00014 9291723

[10] De Graaf R, Kessler RC, Fayyad J, et al The prevalence and effects of adult attention-deficit/hyperactivity disorder (ADHD) on the performance of workers: Results from the WHO World Mental Health Survey Initiative. Occup Environ Med. 2008;65(12):835–842. 10.1136/oem.2007.038448 18505771 PMC2665789

[11] Barkley RA Global issues related to the impact of untreated attention-deficit/hyperactivity disorder from childhood to young adulthood. J Postgrad Med. 2012;120(3):48–59. 10.3810/pgm.2008.09.1907 18824825

[12] Swensen AR, Birnbaum HG, Secnik K, Marynchenko M, Greenberg P, Claxton A Attention-deficit/hyperactivity disorder: Increased costs for patients and their families. J Am Acad Child Adolesc Psychiatry. 2003;42(12):1415–1423. 10.1097/00004583-200312000-00008 14627876

[13] Secnik K, Swensen A, Lage MJ Comorbidities and costs of adult patients diagnosed with attention-deficit hyperactivity disorder. PharmacoEconomics. 2005;23(1):93–102. 10.2165/00019053-200523010-00008 15693731

[14] Bolea-Alamanac B, Nutt DJ, Adamou M, et al 2014. Evidence-based guidelines for the pharmacological management of attention deficit hyperactivity disorder: Update on recommendations from the British Association for Psychopharmacology. J Psychopharmacol. 2014;28(3):179–203. 10.1177/0269881113519509 24526134

[15] National Institute for Health and Clinical Excellence (NICE) Attention deficit hyperactivity disorder. Diagnosis and management of ADHD in children, young people and adults [homepage on the Internet]. 2013 [cited 2013 June 24]. Available from: http://www.nice.org.uk/nicemedia/live/12061/42059/42059.pdf

[16] Canadian Attention Deficit Hyperactivity Disorder Resource Alliance (CADDRA) Canadian ADHD practice guidelines. Toronto, ON: CADDRA; 2011.

[17] Flicker L The influence of opinion leaders. Australian Prescriber. 2012;35(3):74–75. https://doi.org/10.18773/austprescr.2012.031

[18] Health Connexions Key opinion leaders [homepage on the Internet]. 2011 [cited 2013 Aug 5]. Available from: http://healthconnexions.com/blog/market-research/key-opinion-leaders

[19] PRWeb What makes a key opinion leader a KOL? [homepage on the Internet]. 2013 [cited 2013 Jul 17]. Available from: http://www.prweb.com/releases/2012/3/prweb9250355.htm

[20] Atlas.ti Atlas.ti (version 7) [homepage on the Internet]. 2013 [cited 2013 Jun 04]. Available from: www.atlasti.com

[21] Ritchie J, Spencer L Qualitative data analysis for applied policy research In: Bryman A, Burgess R, editors Analysing qualitative data. London: Routledge, 1993; p. 305–329.

